# Associations between visceral adipose and renal artery calcification: Results from the multi-ethnic study of atherosclerosis

**DOI:** 10.1016/j.ajpc.2025.100979

**Published:** 2025-04-02

**Authors:** Harsimran Bajwa, Michael Criqui, Ron Blankstein, Siddique Abbasi, Joao Lima, Jingzhong Ding, Tara Shrout Allen, Matthew Allison

**Affiliations:** a UCSD School of Medicine, 9500 Gilman Drive, La Jolla, CA, 92093, USA; b Brigham and Women's Hospital, 75 Francis Street, Boston, MA, 02115, USA; c Global Development, Amgen, 1 Amgen Center, Newbury Park, CA, 91320, USA; d Department of Cardiology, Johns Hopkins University, 1800 Orleans Street, Baltimore, MD, 21287, USA; e Wake Forest School of Medicine, 475 Vine Street, Winston-Salem, NC, 27101, USA; f San Diego VA Healthcare System, 3350 La Jolla Village Drive, San Diego, CA, 92161, USA

**Keywords:** Renal artery atherosclerosis, Renal artery calcification, Subclinical atherosclerosis, Visceral adipose, Visceral body fat

## Abstract

**Background:**

Visceral adipose tissue (VAT) has been associated with higher levels of atherosclerosis. Renal artery calcification (RAC) secondary to atherosclerosis has been found to be associated with an increase in all-cause mortality.

**Methods:**

1 978 participants underwent CT imaging to measure VAT and RAC. Rate ratio regression was used to estimate prevalence ratios (PRs) for the presence of RAC, while linear regression was used to estimate linear coefficients for the severity of RAC.

**Results:**

1 196 participants had complete VAT and RAC measurements. In adjusted models, VAT area was not associated with RAC presence (PR 1.02, 95 % CI 0.89, 1.16, *p* = 0.80), while greater VAT density was inversely, but not significantly, associated with RAC presence (PR 0.89, 95 % CI 0.78, 1.02, *p* = 0.10). Among 354 participants with RAC > 0, VAT area was significantly associated with RAC severity (slope 63.32, 95 % CI 11.84, 114.81, *p* = 0.02), while VAT density was not associated (slope 9.78, 95 % CI -40.87, 60.44, *p* = 0.71).

**Conclusions:**

VAT area and density are not significantly associated with RAC presence, while greater VAT area is significantly associated with RAC severity among those with RAC > 0. Our results are the first describing the relationship between VAT and RAC, and are in contrast to previous literature demonstrating a significant association between VAT and coronary artery calcification.

## Highlights

**What is already known about this subject?**.

• Visceral adipose tissue has been associated with higher levels of atherosclerosis.
• Renal artery calcification secondary to atherosclerosis has been found to be associated with an increase in all-cause mortality.

**What are the new findings in your manuscript?**.

• Presence of renal artery calcification may be associated with low density visceral adipose.
• Severity of renal artery calcification is associated with high visceral adipose area.

**How might your results change the direction of research or the focus of clinical practice?**.

• Research on visceral adiposity should include measures of adipose density in addition to adipose area as the two may have inverse associations with outcomes.

## Central illustration

**Figure:**
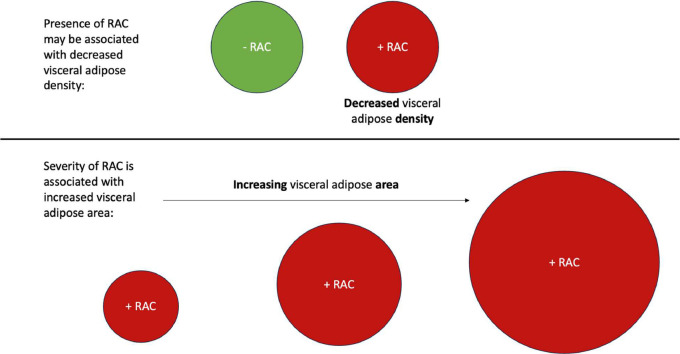

## 1 Introduction

The prevalence of obesity continues to rise, with the most recent age-adjusted estimate being 41.9 % of US adults aged 20 years or older [1]. Obesity related conditions, including cardiovascular disease (CVD), remain leading causes of mortality [2]. In this regard, Burke et al. [3] found that obesity was associated with a greater risk of coronary artery calcium, as well as a greater risk of internal and common carotid intimal medial thickness greater than the 80th percentile.

In populations with and without obesity, the distribution of adipose tissue is relevant to CVD risk since visceral fat has been associated with higher levels of atherosclerosis compared to subcutaneous fat [4]. Recent research has shown that visceral adipose is significantly associated with coronary artery calcium, coronary stenosis, and noncalcified coronary plaques, independent of traditional cardiovascular risk factors [5]. In addition, visceral adipose has been shown to be associated with abdominal aorta calcification [6]. Physiologically, visceral fat releases fatty acids, vasoactive molecules, and adipokines, with the latter accelerating atherosclerosis to include vascular calcification [7]. Moreover, recent literature has highlighted that in addition to the total amount of visceral adipose tissue, the density of that tissue seen on CT may be an important independent predictor of its metabolic activities and downstream effects on CVD [8]. Analyses using data from the Framingham Heart Study found that despite significant associations between lower CT attenuation of visceral adiposity (i.e. less dense, lipid-rich visceral adipose) and cardiometabolic risk, less dense visceral adipose was significantly associated with lower odds of coronary artery calcifications, potentially due to hypertrophic, fibrotic adipocytes appearing more dense on CT [9,10].

Vascular calcification has been associated with cardiovascular disease (CVD) risk factors including hypertension. For instance, Thomas et al. found that renal artery calcification (RAC) was associated with hypertension even when adjusting for abdominal aortic and descending thoracic aortic calcium [11]. As such, RAC may be linked to local changes in the renal vasculature that are germane to the pathogenesis of hypertension. Moreover, RAC is associated with a nearly 40 % increase in all-cause mortality risk even after adjustment for demographic and CVD risk factors, including the presence of vascular calcification in other locations [12].

Given this background, we tested the hypothesis of significant associations between both visceral fat area and density with the presence and extent of renal artery calcification.

## 2 Methods

### 2.1 Subjects

The study design and recruitment methods of the Multi-Ethnic Study of Atherosclerosis (MESA) have been previously published [13]. In brief, MESA is a longitudinal cohort study of 6 814 community-dwelling participants aged 45–84 years at baseline who self-identified as non-Hispanic White or African, Chinese or Hispanic/Latino American. Individuals with clinical evidence of CVD, history of CVD event, or history of invasive procedure for CVD were excluded from participation. Recruitment occurred at six United States communities between July 2000 and August 2002, and participants returned for four follow-up clinical examinations at approximately 18–24-month intervals. All participants provided written informed consent and the institutional review boards at the participating community sites approved the study design.

At clinic visits 2 and 3, a random subset of 1 978 participants from five of the six MESA field centers were enrolled in an ancillary study to determine the extent of abdominal aortic calcification using computed tomography (CT) [14]. Abdominal imaging from this ancillary study was also used to measure the amount of visceral adiposity and to evaluate the presence and extent of RAC. All participants with complete visceral adiposity and RAC measurements were included in the analysis.

### 2.2 Data collection

At all clinic visits, standardized questionnaires were used to obtain demographic, race/ethnicity, social, and health history data. Physical activity was measured in metabolic equivalent (MET)-minutes per week using a 28-item survey.

Height and weight were obtained using standardized measurements. Body mass index (BMI) was computed from these variables and expressed as kg/m^2^. After sitting at rest for 5 min, systolic and diastolic blood pressures were obtained three times with at least one minute between each measurement. The calculation of blood pressure was based on the average of the second and third of three readings. Hypertension was defined as systolic blood pressure greater than or equal to 140 mm Hg, diastolic blood pressure greater than or equal to 90 mm Hg, or the current use of an antihypertensive medication [15].

### 2.3 Laboratory

At all clinic examinations, blood samples were obtained following a 12-hour fast. At visit 2 or 3, and corresponding to the visit at which participants underwent their abdominal CT scan, these blood samples were assayed for total and high-density lipoprotein (HDL) cholesterol, triglycerides, glucose, creatinine levels, C-reactive protein (CRP), Interleukin-6 (IL-6), fibrinogen, leptin, adiponectin, TNF-alpha, and resistin. High-sensitivity CRP and fibrinogen were measured by immune-nephelometry using the BNII instrument. IL-6 was measured by ultrasensitive ELISA. Leptin, adiponectin, TNF-alpha, and resistin were measured using Bio-Rad Luminex flow cytometry.

Diabetes was defined as fasting glucose greater than or equal to 126 mg/dL or the use of hypoglycemic medication. Dyslipidemia was defined as total cholesterol:HDL ratio greater than 5.0 or if the participant used medication to reduce cholesterol. Estimated glomerular filtration rate (eGFR) was calculated using the Chronic Kidney Disease Epidemiology Collaboration equation [16].

### 2.4 Imaging

Participants underwent computed tomography scanning for abdominal aortic calcification at visit 2 or 3. Electron-beam CT scanners were used at two field centers and multidetector CT mode scanners were used at the remaining three field centers. Images were reconstructed in a 35-cm field of view with 5-mm slice thickness. All scan scores were brightness adjusted with a standard phantom.

Six transverse cross-sectional slices of data were interrogated: two at L2–3, two at L3–4, and two at L4–5. Fat tissue was identified as being between −190 and −30 Hounsfield units in density. This definition was chosen based on existing literature that has validated this range in quantifying adipose tissue from CT scans [17]. Visceral adiposity was defined as the fat contained in the visceral cavity. Total visceral fat area was calculated using the sum of visceral fat area over all six available slices. Fat area was indexed to height in meters. Inter-rater and intra-rater reliabilities for total abdominal and visceral cavity areas were 0.99 for all measures. Visceral fat density was defined using the Hounsfield unit values. That is, more negative Hounsfield unit values indicate less dense fat. For example, a Hounsfield unit value of −140 is less dense than a value of −50.

Due to the size of the field of view used for the CT imaging, the positioning of the subject in the scanner, or the size of the subject, parts of the abdomen for some subjects was outside of the field of view and the affected anatomic data could not be processed. In these cases, previously published methods of imputation for missing data were employed [18].

RAC was assessed by summing calcification scores from the renal ostia (right and left) and renal artery (right and left and proximal, middle, and distal third). In keeping with the previously described Agatston scoring method, presence of calcification was identified as a plaque of ≥ 1 mm^2^ with a density of greater than 130 Hounsfield units and severity was based on higher total calcification scores after summing scores from each CT slice [19].

### 2.5 Statistical analysis

The individual, unadjusted associations between visceral adiposity and potential confounders (e.g., demographic characteristics, physical activity, smoking) were assessed by analysis of variance (ANOVA) for continuous variables and overall Chi-square tests for categorical variables.

All CT derived variables were tested for normality. Visceral adipose area and density are normally distributed. For RAC, there were two categories of outcomes: RAC prevalence, which is dichotomous, and the burden of RAC for those with any RAC, which is continuous and normally distributed.

Rate ratio regression was used to estimate the prevalence ratio of RAC as a function of visceral adiposity. Although rate ratio regression was first proposed in the context of cohort studies with common outcomes, the method is also applicable to the estimation of relative prevalences (RPs) in cross-sectional studies [20]. We constructed an unadjusted model (model 1) and models adjusted for confounders. Covariates controlled for in models 2 and 3 were selected based on prior studies identifying associations with both visceral adiposity and arterial calcification [14,21]. Specifically, model 2 was adjusted for sociodemographic characteristics, health behaviors, and clinical markers, including systolic and diastolic blood pressure, total, HDL, and LDL cholesterol, diabetes, and use of aspirin, hypertension medication, and lipid lowering medications. Model 3 controlled for all the same factors as model 2 and included covariates for the adipokines listed in Table 1, including C-reactive protein, interleukin-6, fibrinogen, leptin, adiponectin, TNF-alpha, and resistin.

**Table 1:** Cohort characteristics stratified by quartile of visceral adiposity.

Characteristic	Visceral Adiposity Area 1st Quartile (*n* = 307)	Visceral Adiposity Area 2nd Quartile (*n* = 301)	Visceral Adiposity Area 3rd Quartile (*n* = 309)	Visceral Adiposity Area 4th Quartile (*n* = 279)	P-Value (Area)	Visceral Adiposity Density 1st Quartile (lowest density) (*n* = 248)	Visceral Adiposity Density 2nd Quartile (*n* = 306)	Visceral Adiposity Density 3rd Quartile (*n* = 315)	Visceral Adiposity Density 4th Quartile (highest density) (*n* = 327)	P-Value (Density)
Renal Artery Calcium Present, n ( %)	67 (21.82 %)	79 (26.25 %)	104 (33.66 %)	104 (37.28 %)	< 0.01	73 (29.44 %)	105 (34.31 %)	87 (27.62 %)	89 (27.22 %)	0.19
Age (years), mean (SD)	64.43 (10.02)	65.30 (9.65)	66.356 (9.10)	68.15 (9.46)	< 0.01	65.60 (8.87)	66.33 (9.62)	65.36 (9.65)	66.65 (10.22)	0.39
Gender, n ( %)										
Female	219 (71.33 %)	181 (60.13 %)	156 (50.49 %)	108 (38.71 %)	< 0.01	113 (45.56 %)	137 (44.77 %)	139 (44.13 %)	143 (43.73 %)	0.97
Race*, n ( %)										
White	114 (37.13 %)	90 (29.90 %)	111 (35.92 %)	117 (41.94 %)	< 0.01	108 (43.55 %)	108 (35.29 %)	104 (33.02 %)	112 (34.25 %	< 0.01
Chinese	60 (19.54 %)	56 (18.60 %)	36 (11.65 %)	16 (5.73 %)		6 (2.42 %)	39 (12.75 %)	64 (20.32 %)	59 (18.04)	
Black	81 (26.38 %)	81 (26.91 %)	56 (18.12 %)	45 (16.13 %)		30 (12.10 %)	68 (22.22 %)	63 (20.00 %)	102 (31.19 %)	
Hispanic/Latino	52 (16.94 %)	74 (24.58 %)	106 (34.30 %)	101 (36.20 %)		104 (41.94 %)	91 (29.74 %)	84 (26.67 %)	54 (16.51 %)	
Total gross family income, n ( %)										
$49,999 or below	179 (58.31 %)	191 (63.46 %)	192 (62.14 %)	191 (68.46 %)	0.09	167 (67.34 %)	201 (65.69 %)	187 (59.37 %)	198 (60.55 %)	0.13
$50,000 or above	128 (41.69 %)	110 (36.54 %)	117 (37.86 %)	88 (31.54 %)		81 (32.66 %)	105 (34.31 %)	128 (40.63 %)	129 (39.45 %)	
Education, n ( %)										
High school grad or less	94 (30.62 %)	121 (40.20 %)	114 (36.89 %)	121 (43.37 %)	0.03	113 (45.56 %)	117 (38.24 %)	122 (38.73 %)	98 (29.97 %)	< 0.01
Some college	98 (31.92 %)	78 (25.91 %)	99 (32.04 %)	80 (28.67 %)		76 (30.65 %)	93 (30.39 %)	78 (24.76 %)	108 (33.03 %)	
College grad or above	115 (37.46 %)	102 (33.89 %)	96 (31.07 %)	78 (27.96 %)		59 (23.79 %)	96 (31.37 %)	115 (36.51 %)	121 37.00 %)	
BMI (kg/m^2^), mean (SD)	23.89 (3.76)	27.22 (4.62)	28.86 (4.09)	32.10 (4.90)	< 0.01	31.99 (5.09)	29.25 (4.86)	27.16 (4.24)	24.36 (3.76)	< 0.01
Smoking status, n ( %)										
Never	145 (47.70 %)	162 (54.18 %)	146 (47.40 %)	109 (39.64 %)	< 0.01	118 (47.58 %)	147 (48.04 %)	162 (51.43 %)	135 (41.28 %)	0.05
Former	119 (39.14 %)	101 (33.78 %)	136 (44.16 %)	142 (51.64 %)		108 (43.55 %)	130 (42.48 %)	117 (37.14 %)	143 (43.73 %)	
Current	40 (13.03 %)	36 (12.04 %)	26 (08.44 %)	24 (08.73 %)		17 (6.85 %)	29 (9.48 %)	35 (11.11 %)	45 (13.76 %)	
Moderate to vigorous physical activity (min/week), mean (SD)	4776.18 (4458.26)	4728.30 (4590.19)	4867.87 (4449.66)	4293.85 (4561.46)	0.29	5078.92 (4522.73)	4442.70 (4820.04)	4489.51 (4386.62)	4769.71 (4320.96)	0.57
Sedentary activity (min/week), mean (SD)	1622.73 (1063.93)	1691.86 (1034.03)	1680.97 (1113.77)	1904.05 (1135.48)	< 0.01	1807.20 (1220.89)	1779.32 (1057.29)	1686.66 (1070.08)	1632.78 (1030.87)	0.03
Diabetes, n ( %)	16 (05.25 %)	41 (13.62 %)	54 (17.48 %)	63 (22.58 %)	< 0.01	50 (20.16 %)	46 (15.03 %)	42 (13.33 %)	36 (11.01 %)	0.02
Dyslipidemia, n ( %)	60 (20.13 %)	106 (36.18 %)	134 (44.52 %)	146 (52.71 %)	< 0.01	127 (51.21 %)	137 (44.77 %)	107 (33.97 %)	75 (22.94 %)	< 0.01
Hypertension, n ( %)	142 (47.33 %)	184 (38.05 %)	200 (65.57 %)	219 (79.06 %)	< 0.01	171 (68.95 %)	207 (67.65 %)	189 (60.00 %)	178 (54.43 %)	< 0.01
C-reactive protein (mg/L), mean (SD)	2.37 (7.71)	3.22 (7.17)	3.25 (5.08)	4.58 (10.93)	< 0.01	3.92 (4.78)	4.18 (10.89)	2.76 (4.27)	2.61 (9.22)	< 0.01
Interleukin-6 (pg/mL), mean (SD)	1.91 (1.78)	2.18 (1.63)	2.46 (1.67)	3.10 (1.80)	< 0.01	2.94 (1.83)	2.64 (1.81)	2.11 (1.45)	2.04 (1.84)	< 0.01
Fibrinogen (mg/dL), mean (SD)	417.24 (81.49)	441.20 (91.31)	442.85 (80.04)	455.08 (91.53)	< 0.01	451.19 (83.98)	449.72 (89.11)	440.09 (83.33)	417.63 (87.48)	< 0.01
Leptin (ng/mL), mean (SD)	13.56 (14.18)	21.13 (22.14)	24.29 (26.06)	28.20 (24.65)	< 0.01	28.20 (24.65)	24.49 (26.06)	21.13 (22.14)	13.56 (14.18)	< 0.01
Adiponectin (μg/mL), mean (SD)	29.68 (17.50)	20.39 (11.29)	18.81 (12.02)	17.28 (11.03)	< 0.01	17.52 (9.83)	18.92 (12.99)	20.01 (11.58)	28.84 (17.20)	< 0.01
TNF-alpha (pg/mL), mean (SD)	6.01 (15.44)	5.47 (6.49)	5.73 (4.22)	5.80 (3.60)	0.87	6.34 (6.45)	5.22 (3.25)	5.12 (3.46)	6.41 (15.36)	0.80
Resistin (ng/mL), mean (SD)	15.47 (7.29)	16.55 (7.50)	16.80 (13.77)	17.53 (7.08)	< 0.01	16.76 (6.23)	16.89 (7.50)	15.60 (6.68)	17.05 (14.17)	0.91

Linear regression was used to estimate the relationship between visceral adiposity and the severity of renal artery calcification among participants with RAC > 0. We utilized the same modeling strategy as described above.

Unadjusted models assessing the interaction between visceral adiposity area and density were constructed for rate ratio regression. When interaction terms were significant, we constructed stratified models.

Two-tailed p-values of < 0.05 were considered statistically significant. All statistical analyses were conducted using R software for statistical computing [22].

## 3 Results

Of the 1 978 participants enrolled in the abdominal CT ancillary study, 1923 had visceral adiposity measurements. Of the latter, 1196 participants had CT scans that continued cephalad enough to contain the renal arteries and assess for the presence of RAC. Among these, the average age at time of CT scan was 66 years (SD 9.6); 55 % were female, 36 % were non-Hispanic White, 14 % were Chinese American, 22 % were African American, and 28 % were Hispanic/Latino American.

As shown in Table 1 and compared to individuals in the lowest quartile of total visceral adiposity area, participants in the higher three quartiles were significantly more likely to have a RAC score > 0 (*p* < 0.01). This trend was not seen across quartiles of visceral adiposity density. The first, or least dense, quartile of visceral adiposity density corresponded to < −95.88 Hounsfield units, the second quartile corresponded to −95.88 to −90.875 Hounsfield units, the third quartile corresponded to −90.875 to −85.005 Hounsfield units, and the fourth, or most dense, quartile corresponded to > −85.005 Hounsfield units. Participants in the highest quartile of visceral adiposity area or the first quartile of visceral adiposity density were also significantly more likely to be White or Hispanic/Latino American, have a higher BMI, and to not have gone to college (Table 1). In addition, participants in the second, third, and fourth quartiles of visceral adiposity area were significantly more likely to be older, male, and a former or current smoker in comparison to participants in the lowest quartile of visceral adiposity area. These characteristics were not significantly associated with visceral adiposity density.

Table 2 displays the results of rate ratio regression analyses examining the independent association of standard deviation increments of both visceral adiposity area and density with the presence of RAC. In model 1, a 1-standard deviation increment in visceral adiposity area was associated with a 18 % higher prevalence of RAC (95 % CI 1.09 – 1.27, *p* < 0.01), while this increment of visceral adiposity density was associated with an 8 % lower prevalence of RAC (95 % CI 0.85 – 1.00, *p* = 0.06). After adjustment for the variables in model 2, the associations between visceral adiposity area and presence of RAC were no longer statistically significant. In contrast, the relationship between visceral adiposity density and the presence of RAC was statistically significant (0.89, 0.80 – 0.99, 0.04). In model 3, the prevalence ratio of RAC for a one standard deviation increment in visceral adiposity area was 1.08 (0.97 – 1.21, 0.16), and the prevalence ratio of RAC for a one standard deviation increment in visceral adiposity density was 0.88 (0.79 – 0.99, 0.04).

**Table 2:** Rate ratio regression analyses of the association of visceral adiposity area and density and presence of RAC.

Model	Relative Prevalence⁎⁎	95 % Confidence Interval	P-Value
1			
Visceral adiposity area alone	1.18	1.09 – 1.27	0.00
Visceral adiposity area in combined model*	1.26	1.12 – 1.41	0.00
Visceral adiposity density alone	0.92	0.85 – 1.00	0.06
Visceral adiposity density in combined model*	1.10	0.97 – 1.25	0.15
2			
Visceral adiposity area alone	1.08	0.97 – 1.20	0.16
Visceral adiposity area in combined model	1.01	0.89 – 1.15	0.83
Visceral adiposity density alone	0.89	0.80 – 0.99	0.04
Visceral adiposity density in combined model*	0.90	0.79 – 1.02	0.10
3			
Visceral adiposity area alone	1.08	0.97 – 1.21	0.16
Visceral adiposity area in combined model	1.02	0.89 – 1.16	0.80
Visceral adiposity density alone	0.88	0.79 – 0.99	0.04
Visceral adiposity density in combined model*	0.89	0.78 – 1.02	0.10

Model 1: Unadjusted.

Model 2: Adjusted for sex, age, BMI, race, income, education, smoking history, physical activity, sedentary behavior, systolic blood pressure, diastolic blood pressure, use of hypertension medication, total cholesterol, HDL cholesterol, LDL cholesterol, use of lipid-lowering medication, use of diabetes medication, use of aspirin.

Model 3: Adjusted for sex, age, BMI, race, income, education, smoking history, physical activity, sedentary behavior, systolic blood pressure, diastolic blood pressure, use of hypertension medication, total cholesterol, HDL cholesterol, LDL cholesterol, use of lipid-lowering medication, use of diabetes medication, use of aspirin, and adipokine levels.

⁎ “Combined model” indicates that visceral adipose area and density were both included as predictor variables.

⁎⁎ Relative prevalence of visceral adiposity per 1 standard deviation increase in visceral adiposity area and/or density.

In an unadjusted model where both visceral adiposity area and density were included as predictors (“combined model”), the relationship between visceral adiposity area and presence of RAC remained significant (1.26, 1.12 – 1.41, 0.01), while the association of visceral adiposity density with the presence of RAC was not significant (1.10, 0.97 – 1.25, 0.15). However, in the “combined” models 2 and 3, neither visceral adiposity area nor density were significantly associated with presence of RAC (model 2 area: 1.01, *p* = 0.83; model 2 density: 0.90, *p* = 0.10; model 3 area: 1.02, *p* = 0.80; model 3 density: 0.89, *p* = 0.10).

Table 3 displays the estimates, 95 % confidence intervals, and p-values for the linear regression models estimating the relationship between visceral adiposity area and density and the severity of RAC among the 354 participants with RAC > 0. In model 1, a 1-standard deviation increment in visceral adiposity area was associated with a 37.91 increase in total RAC Agatston score (95 % CI 11.5 – 64.4, *p* = 0.01), while a 1-standard deviation increment in visceral adiposity density was associated with a 10.50 decrease in total RAC Agatston score (95 % CI −38.58 – 17.57, *p* = 0.46). In models 2 and 3, the results were similar.

**Table 3:** Linear regression analysis of the association between visceral adiposity area and density and amount of RAC, among those with RAC.

Model	Estimate (Slope)⁎⁎	95 % Confidence Interval	P-Value
1			
Visceral adiposity area alone	37.91	11.47 – 64.35	0.01
Visceral adiposity area in combined model*	57.14	21.42 – 92.87	0.00
Visceral adiposity density alone	−10.50	−38.58 – 17.57	0.46
Visceral adiposity density in combined model*	29.98	−7.56 – 67.52	0.11
2			
Visceral adiposity area alone	58.06	19.00 – 97.12	0.00
Visceral adiposity area in combined model*	62.39	13.86 – 110.92	0.01
Visceral adiposity density alone	−28.66	−66.87 – 9.55	0.14
Visceral adiposity density in combined model*	7.08	−39.90 – 54.06	0.77
3			
Visceral adiposity area alone	57.62	15.51 – 99.72	0.01
Visceral adiposity area in combined model*	63.32	11.84 – 114.81	0.02
Visceral adiposity density alone	−25.96	−67.81 – 15.90	0.23
Visceral adiposity density in combined model*	9.78	−40.87 – 60.44	0.71

Model 1: Unadjusted.

Model 2: Adjusted for sex, age, BMI, race, income, education, smoking history, physical activity, sedentary behavior, systolic blood pressure, diastolic blood pressure, use of hypertension medication, total cholesterol, HDL cholesterol, LDL cholesterol, use of lipid-lowering medication, use of diabetes medication, use of aspirin.

Model 3: Adjusted for sex, age, BMI, race, income, education, smoking history, physical activity, sedentary behavior, systolic blood pressure, diastolic blood pressure, use of hypertension medication, total cholesterol, HDL cholesterol, LDL cholesterol, use of lipid-lowering medication, use of diabetes medication, use of aspirin, and adipokine levels.

⁎ “Combined model” indicates that total visceral adipose area and density were both included as predictor variables.

⁎⁎ Estimate of change in amount of RAC per 1 standard deviation increase in visceral adiposity area and/or density.

When both area and density were in the same model 1, visceral adiposity area remained a significant predictor (*p* < 0.01) while the association with visceral adiposity density remained nonsignificant (*p* = 0.11). In model 3, a 1-standard deviation increment in visceral adiposity area was associated with a 63.32 increase in total RAC Agatston score (95 % CI 11.84 – 114.81, *p* = 0.02) even when controlling for visceral adiposity density. Visceral adiposity density continued to be nonsignificant in the combined model 3 (estimate: 9.78, 95 % CI −40.87 – 60.44, *p* = 0.71).

There were statistically significant interactions between visceral adiposity area (dichotomous) and density (continuous) predicting RAC presence (*p* = 0.03), as well as area (continuous) and density (dichotomous), (*p* < 0.01) (Table 4). Specifically, visceral fat area had a stronger positive association for RAC presence when visceral fat density was higher, i.e. more dense (1.25, 0.99 - 1.59, *p* = 0.06) than when visceral fat density was less dense (0.92, 0.79 – 1.07, *p* = 0.29). Similarly, visceral fat density had a stronger association when visceral fat area was below the 50th percentile (0.92, 0.73 - 1.16, *p* = 0.50) than when visceral fat area was above the 50th percentile (1.01, 0.87 – 1.10, *p* = 0.91). There were no significant interactions between visceral adiposity area and density predicting RAC severity.

**Table 4:** Interaction analyses between visceral adiposity area and density on RAC presence.

Interaction Model	PR	95 % Confidence Interval	P-Value
Visceral adiposity area (continuous) and visceral adiposity density (binary)			0.00
Visceral adiposity area alone, stratified to low* visceral adiposity density	0.92	0.79 – 1.07	0.29
Visceral adiposity area alone, stratified to high* visceral adiposity density	1.25	0.99 – 1.59	0.06
Visceral adiposity density (continuous) and visceral adiposity area (binary)			0.03
Visceral adiposity density alone, stratified to low* visceral adiposity area	0.92	0.73 – 1.16	0.50
Visceral adiposity density alone, stratified to high* visceral adiposity area	1.01	0.87 – 1.10	0.91

⁎ Low refers to participants in the first and second quartiles, high refers to participants in the third and fourth quartiles.

## 4 Discussion

In this relatively large multi-ethnic cohort of adults from five communities across the United States, we demonstrated that visceral adiposity area and density are not significantly associated with the presence of RAC. Increasing visceral adiposity area was significantly associated with higher levels of calcification among individuals with any RAC, while visceral adiposity density was not significant with RAC burden.

While the unadjusted association between visceral adiposity area and presence RAC was highly significant in model 1, this relationship was largely attenuated by the addition of sociodemographic characteristics and CVD risk factors in model 2 and suggests that these variables mediate the association between visceral adiposity and atherosclerosis of the renal arteries. In contrast, among individuals with RAC > 0, total visceral adiposity area was a significant predictor of severity of calcification. It is well understood that unresolved inflammation at the site of arterial injury is capable of propagating foam cell and fatty streak formation. Therefore, it may be possible that among individuals with RAC > 0, additional visceral adiposity area contributes to greater arterial inflammation and therefore increased severity of atherosclerosis [23]. The addition of adipokines in model 3 did not significantly alter either of these relationships, making it unlikely that adipokines were mediators for visceral fat.

After controlling for sociodemographic characteristics, CVD risk factors, adipokines, and visceral adiposity area, higher density visceral adipose had an inverse association with RAC presence, however this relationship was not significant. Visceral adiposity density was not a significant predictor for severity of RAC among those with RAC > 0 in any model. Prior human studies have demonstrated that higher density adipose is positively associated with mortality, but not with systemic inflammation [24]. As such, dense visceral adipose may lack the effect on systemic inflammation necessary to drive plaque progression to severe vascular calcification.

In unadjusted models evaluating the interaction between visceral adiposity area and density, there was no interaction effect for RAC severity, but there was significant interaction for RAC presence. Visceral adiposity area had a stronger positive association with RAC presence when visceral adipose was denser, while visceral adiposity density had a stronger inverse association with RAC presence when there was less visceral adiposity area. These findings allude to the close relationship between fat quantity and quality, whereby change in one factor at a time demonstrated stronger individual associations with RAC presence, while simultaneous increase of visceral adiposity area and decrease of visceral adiposity density, associated with general pathogenic adipose remodeling, masks individual associations.

Ding et al. [25] previously examined the association between non-subcutaneous adiposity and calcified coronary plaques, and similar to our results, their findings demonstrated a statistically significant unadjusted association between volume of abdominal visceral fat and presence of calcified coronary plaques that was attenuated upon adjustment for CVD risk factors, including systolic blood pressure, total HDL cholesterol, and diabetes status. However, Ohashi et al. [5] and Haidar et al. [26] found significant associations between visceral adiposity and coronary artery calcification and noncalcified coronary plaques, even after adjusting for traditional cardiovascular risk factors. It remains unclear what is driving the discrepancy between the previously reported association between visceral adiposity area or density and coronary artery calcium; our findings that visceral adiposity is not significantly associated with RAC presence diverge. One possible explanation is that the pathogenesis of coronary artery calcium and renal artery calcium are driven by slightly different factors. RAC is highly correlated with abdominal aorta calcification, and in an analysis of MESA data, Criqui et al. [14] found that in comparison to abdominal aorta calcification, coronary artery calcification had weaker associations with cardiovascular risk factors such as smoking and dyslipidemia [27]. This may explain why controlling for these factors attenuated the relationship between visceral adiposity and RAC, but did not change the significance of the association between visceral adiposity and coronary artery calcification. Another possibility is that coronary artery calcification is predominately intimal calcification associated with atherosclerosis, while RAC is more likely to include arterial media calcification, which is associated with additional etiologies other than atherosclerosis [28,29].

Although our results were not statistically significant, the inverse association that we observed between visceral adiposity density and RAC, whereby increasing visceral adiposity density was associated with lower prevalence and burden of RAC, is congruent with recent research. Lee et al. [8] found that increasing visceral fat area and decreasing fat density was significantly associated with hypertension, hypercholesterolemia, hypertriglyceridemia, diabetes, and metabolic syndrome beyond the associations with generalized adiposity. Haidar et al. [26] found that higher density visceral adiposity on CT (i.e. less lipid-dense fat tissue) was associated with decreased likelihood of having a coronary artery calcium score > 0.

Our study advances the literature on RAC in several ways. First, this is the first study to examine the relationship between visceral adipose tissue and RAC. Prior literature had largely focused on the association of visceral adiposity and calcification of the coronary arteries or the abdominal aorta. In addition, we have shown that visceral adiposity area is a predictor of the severity of RAC among those with RAC > 0, even when adjusting for sociodemographic characteristics, CVD risk factors, and visceral adiposity density. Lastly, we have found that adipokines do not appear to be mediating the relationship between visceral adipose and RAC.

Due to the cross-sectional nature of this analysis, causal associations cannot necessarily be inferred. In addition, many patients without sufficient CT visualization of the renal arteries were excluded from this study and may have introduced bias. Also, our analyses utilized Agatston scoring for quantification for RAC. As Criqui et al. [30] reported, Agatston scoring alone is not optimal for CVD risk prediction. Therefore, our findings on the association between visceral adiposity area and density and RAC should not be extrapolated to CVD risk at large. Lastly, the study population consisted of adults aged 45–84 years without clinically apparent CVD at baseline. Given this, our findings may not be generalized to secondary prevention populations.

Given previous studies that have established links between RAC and hypertension as well as RAC and increased all-cause mortality independent of atherosclerosis in other vascular beds, our findings suggest that adults with elevated visceral adiposity area may be at higher risk of morbidity related to more severe RAC [12,31].

## 5 Author agreement form

This statement is to certify that all authors have seen and approved the manuscript being submitted, have contributed significantly to the work, attest to the validity and legitimacy of the data and its interpretation, and agree to its submission to the *American Journal of Preventive Cardiology.*

We attest that the article is the Authors' original work, has not received prior publication and is not under consideration for publication elsewhere.

On behalf of all Co-Authors, the corresponding Author shall bear full responsibility for the submission. Any changes to the list of authors, including changes in order, additions or removals will require the submission of a new author agreement form approved and signed by all the original and added submitting authors.

## CRediT authorship contribution statement

**Harsimran Bajwa:** Writing – review & editing, Writing – original draft, Project administration, Formal analysis, Data curation, Conceptualization. **Michael Criqui:** Writing – review & editing, Writing – original draft, Supervision, Methodology, Investigation, Conceptualization. **Ron Blankstein:** Writing – review & editing, Writing – original draft. **Siddique Abbasi:** Writing – review & editing, Writing – original draft. **Joao Lima:** Writing – review & editing, Writing – original draft. **Jingzhong Ding:** Writing – review & editing, Writing – original draft. **Tara Shrout Allen:** Writing – review & editing. **Matthew Allison:** Writing – review & editing, Writing – original draft, Supervision, Methodology, Formal analysis, Conceptualization.

## Declaration of competing interest

The authors declare that they have no known competing financial interests or personal relationships that could have appeared to influence the work reported in this paper.

## Appendix Supplementary materials

Image, application 1
