# Supplementary material for: Associations between visceral adipose and renal artery calcification: Results from the multi-ethnic study of atherosclerosis
Source: Am J Prev Cardiol. 2025 Apr 2;22:100979. doi: 10.1016/j.ajpc.2025.100979 (PMC12017846; doi:10.1016/j.ajpc.2025.100979)
Supplement: Supplementary file 1 [file mmc1.docx]

**Result of unadjusted rate ratio regression analyses assessing the relationship between VAT area and density quartiles with RAC presence:**

VAT area quartiles and RAC presence: PR 1.10, p-value 0.07

VAT density quartiles and RAC presence PR 1.05, p-value 0.21

**Additional background literature on the relationship between VAT and coronary artery calcification:**

Ohashi N, Yamamoto H, Horiguchi J, et al. Visceral fat accumulation as a predictor of coronary artery calcium as assessed by multislice computed tomography in Japanese patients. *Atherosclerosis*. 2009;202(1):192-199. doi:[10.1016/j.atherosclerosis.2008.04.019](https://doi.org/10.1016/j.atherosclerosis.2008.04.019)

Abstract: The impact of visceral adiposity on subclinical coronary atherosclerosis is unclear in Japanese patients. We investigated the sex-specific relationship between the amount of visceral fat and coronary artery calcium (CAC) using multislice computed tomography (MSCT). This is a cross-sectional study of 321 consecutive Japanese patients (213 men and 108 women) who underwent MSCT scanning for the examination of coronary heart disease. CAC score, visceral fat area (VFA), subcutaneous fat area (SFA), and waist circumference (WC) were determined by MSCT for all patients. The prevalence of detectable CAC was 73% and 57% in men and women, respectively. Using a multivariable logistic and ordinal regression analyses adjusting for traditional cardiovascular risk factors and adiposity measurements, VFA represented an independent predictor of the presence and extent of CAC (odds ratio (95% confidence interval) per one-unit-standard deviation increase in VFA: 2.48 (1.23–6.05) in logistic regression analysis; 2.05 (1.18–3.98) in ordinal regression analysis). Similar relationships were observed across the gender. We further assessed the sex-specific cut-off levels of VFA and WC to predict the presence of CAC. The results of receiver operator characteristic analysis indicated that the VFA cut-off level in men was 116cm^2^; and in women, it was 82cm^2^, corresponding to WC values of 87.7cm in men and 82.6cm in women. In conclusion, we found that visceral adiposity measured by MSCT is significantly associated with the presence and extent of CAC as a marker of subclinical atherosclerosis in Japanese patients.

Ohashi N, Yamamoto H, Horiguchi J, et al. Association between visceral adipose tissue area and coronary plaque morphology assessed by CT angiography. *JACC Cardiovasc Imaging*. 2010;3(9):908-917. doi:[10.1016/j.jcmg.2010.06.014](https://doi.org/10.1016/j.jcmg.2010.06.014)

Abstract: Objectives: We sought to investigate the association between visceral adipose tissue (VAT) with the presence, extent, and characteristics of noncalcified coronary plaques (NCPs) using 64-slice computed tomography angiography (CTA). Background: Although visceral adiposity is associated with cardiovascular events, its association with NCP burden and vulnerability is not well known. Methods: The study population consisted of 427 patients (age 67 ± 11 years; 63% men) with proven or suspected coronary artery disease who underwent 64-slice CTA. We assessed the presence and number of NCPs for each patient. The extent of NCP was tested for the difference between high (≥2) and low (≤1) counts. We further evaluated the vulnerable characteristics of NCPs with positive remodeling (remodeling index >1.05), low CT density (≤38 HU), and the presence of adjacent spotty calcium. Plain abdominal scans were also performed to measure the VAT and subcutaneous adipose tissue area. Results: A total of 260 (61%) patients had identifiable NCPs. Multivariate analyses revealed that increased VAT area (per 1 standard deviation, 58 cm^2^) was significantly associated with both the presence (odds ratio [OR]: 1.68; 95% confidence interval [CI]: 1.28 to 2.22) and extent (OR: 1.31; 95% CI: 1.03 to 1.68) of NCP. Other body composition measures, including subcutaneous adipose tissue area, body mass index, and waist circumference were not significantly associated with either presence or extent of NCP. Increased VAT area was also independently associated with the presence of NCP with positive remodeling (OR: 1.71; 95% CI: 1.18 to 2.53), low CT density (OR: 1.69; 95% CI: 1.17 to 2.47), and adjacent spotty calcium (OR: 1.52; 95% CI: 1.03 to 2.27). Conclusions: Increased VAT area was significantly associated with NCP burden and vulnerable characteristics identified by CTA. Our findings may explain the excessive cardiovascular risk in patients with visceral adiposity, and support the potential role of CTA to improve risk stratification in such patients.
